# MLGT: A multimodal graph attention network for virtual screening of anti—Uveitis drugs

**DOI:** 10.1371/journal.pone.0343159

**Published:** 2026-03-05

**Authors:** Yu Sun, Yihang Qin, Wenhao Chen

**Affiliations:** 1 School of Special Education, Changchun University, China, Changchun; 2 School of Computer Science and Technology, Changchun University, China, Changchun; University of Nebraska Medical Center, UNITED STATES OF AMERICA

## Abstract

Uveitis is a severe ocular inflammatory disease with complex immune—mediated pathogenesis, posing significant challenges for drug discovery. While artificial intelligence has accelerated virtual screening, existing models often inadequately integrate heterogeneous molecular features or address disease—specific mechanisms. To address these gaps, we propose MLGT (Multimodal Learning with Graph and molecular descriptors for Therapeutics), a novel graph attention network based on GATv2 that synergistically integrates molecular graph topology, bond attributes, and physicochemical descriptors within a unified deep learning framework. The model employs dynamic attention mechanisms to capture non—local atomic interactions and a dual—stream fusion module to combine graph embeddings with molecular descriptors. To mitigate data imbalance and overfitting, we implement label smoothing, class—balanced sampling, and SMILES randomization. Evaluated on a rigorously curated Uveitis—related compound dataset from ChEMBL, MLGT achieves state—of—the—art performance: 97.7% accuracy, 97.2% F1 score, 96.1% recall, and an AUC—ROC of 0.9156, surpassing existing graph learning and classical machine learning benchmarks. Ablation studies confirm the essential roles of multimodal fusion and attention mechanisms. This study provides an efficient, attention—based computational tool for targeted Uveitis drug screening and establishes a scalable AI—driven paradigm for precision drug discovery in complex diseases.

## Introduction

Uveitis is an ocular disease that severely impacts vision, with diverse clinical manifestations and complex pathogenesis [1]. It is among the leading causes of blindness worldwide. The pathological mechanisms of Uveitis are highly heterogeneous—reflecting associations with systemic immune disorders, infections, and genetic factors—which complicates diagnosis and treatment. Current clinical management primarily relies on corticosteroids, immunosuppressants, and targeted biologic therapies, but these regimens often carry significant risks of adverse effects and uncertain efficacy [2]. In the context of drug development, traditional wet—lab screening methods are time—consuming and costly, severely limiting the efficiency of translating novel therapies into clinical practice. Therefore, developing rapid, precise, and cost—effective drug screening methods has become a critical and urgent challenge.

Computer—aided drug discovery has emerged as a transformative approach in pharmaceutical research, leveraging computational models to predict molecular properties and accelerate the screening of therapeutic candidates [3]. In particular, AI—driven methods demonstrate extraordinary potential for identifying promising compounds from vast chemical spaces, thus reducing reliance on costly and time—consuming wet—lab experiments [4]. Within this context, the application of machine learning and deep learning to predict drug–target interactions and compound efficacy represents a major breakthrough in rational drug design.

In recent years, rapid advancements in computer science have introduced revolutionary technologies to drug discovery. AI technologies, particularly deep learning, have been widely applied in medicinal chemistry, enabling multi—level optimization from molecular design to drug screening. Cutting—edge research indicates that graph neural networks (GNNs) operating on molecular graphs demonstrate high efficiency and strong generalization capabilities in predicting molecular properties [5].

Simultaneously with the rapid development of graphic neural networks, the paradigm shift in drug discovery in recent years has tended toward more knowledge-intensive and expressive AI frameworks. A prominent direction has been the integration of large pre-trained language models (LLMs) with biomedical knowledge maps and molecular maps. By encoding large biomedical corpus of text and structured domain knowledge, LLM-based models can provide contextual semantic representations that significantly improve prediction accuracy, interpretability, and generalization under limited labeled data. Representative studies such as LLM-DDI show that combining LLM-derived embeddings with graph-based inference, over biomedical knowledge maps, can produce significant performance improvements in drug interaction prediction.

Another influential trend is multi-view contrast learning, which attempts to align heterogeneous drug representation through contrast targets, such as molecular maps, fingerprint maps, drug interaction networks, and physicochemical descriptors. These methods show strong robustness against data scarcity and noise, especially for drug interaction and event-level prediction tasks in pharmacology. Recently, multi-view contrast frameworks for drug-drug interaction modeling have consistently outperformed single-view encoders by enforcing cross-view semantic consistency and representational unwinding.

These emerging paradigms jointly emphasize the importance of utilizing heterogeneous information sources and robust characterization learning strategies in modern drug discovery pipelines. While the LLM-centered approach primarily emphasizes the extraction of knowledge from text and large-scale biomedical cartography, while the contrast framework focuses on cross-perspective alignment, the proposed MLGT framework adopts a chemistry-centered perspective by tightly integrating atomic-level cartographic attention with global physicochemical descriptors under unified learning goals. In this sense, MLGT can be viewed as a complementary, domain-based alternative that is particularly suitable for small molecular screening tasks in limited active samples, such as the discovery of drugs for meningitis.

However, current AI—based drug screening models still face significant limitations. First, much research has focused on broad—spectrum anti—inflammatory or anti—cancer drugs, often lacking precise modeling of specific pathological mechanisms, such as the immune—inflammatory processes underlying Uveitis [6]. Second, traditional approaches typically rely on single—source structural information (e.g., SMILES representations), failing to fully integrate complex molecular graph structures, bond attributes, and descriptor features, thereby limiting improvements in model performance [7]. Additionally, data imbalance and diversity in molecular representations hinder existing models’ effectiveness on small—scale, high—resolution datasets.

Given these limitations, there is an urgent need for an efficient, precise computational approach tailored to the pathological characteristics of Uveitis in order to optimize the drug screening process. Such an approach should transcend the constraints of single—modal representations by comprehensively integrating molecular topological features, bond properties, and descriptor information into a unified multimodal predictive framework. Concurrently, data processing should employ balancing techniques and advanced training strategies to mitigate data imbalance and noise, thereby enhancing the model’s generalization capability and practical applicability.

In this study, we develop a customized GNN—based drug screening model to address key technical bottlenecks in Uveitis drug candidate selection [8]. We propose a framework that integrates the GATv2 (Graph Attention v2) network with multimodal molecular feature representations. By deeply fusing molecular structural information, descriptors, and graph—based features, our approach enables precise screening of therapeutically promising candidates from vast compound libraries. The primary innovations of this work include: First, GATv2 is used to capture complex molecular graph structures and bond features, enabling efficient modeling of intricate molecular topology [9];Second, integrating traditional chemical descriptors with graph—derived features to enhance the model’s predictive accuracy for molecules related to Uveitis mechanisms; Third, optimizing the training process through techniques such as label smoothing and class balancing to effectively mitigate data imbalance [10]; Fourth, exploring data augmentation strategies in molecular screening to improve model adaptability on noisy, low—resource datasets [11]; and Fifth, using strategies like early stopping and learning rate scheduling to optimize training efficiency and performance. This study not only provides an efficient computational framework for Uveitis drug screening but also offers a scalable and transferable AI methodology for targeted therapy development in specific disease contexts.

Through extensive experiments on a carefully curated dataset of Uveitis—related compounds, we demonstrate that our model achieves industry—leading predictive performance, offering a viable computational alternative to traditional drug screening techniques. This study exemplifies how modern AI can address pressing biomedical challenges by enabling the rapid, cost—effective, and precise identification of novel therapeutic compounds with genuine clinical potential.

In this study, we introduced MLGT (Multimodal Learning with Graph and molecular descriptors for Therapeutics), This is a gatv2 based architecture that integrates molecular graph representation with chemically meaningful descriptors through a two stream fusion mechanism. MLGT uses dynamic attention mechanism to adaptively weight the interaction between atoms, and uses descriptor aware graph pool strategy to connect the local topology with the global physical and chemical properties. This design enables the model to capture structural nuances and pharmacological properties, especially the multi—target immune imbalance against Uveitis.

In summary, this work pioneers the use of GNNs in Uveitis drug screening [12]. By integrating multimodal data representations and advanced optimization strategies, our framework significantly enhances the precision and efficiency of the computational drug screening process. In the future, this approach holds promise for broader applications in targeted therapy development for other diseases, thereby advancing the field of precision medicine.

## Related works

### 2.1. Traditional computational biology methods and their limitations

Before the advent of deep learning, drug screening primarily relied on traditional computational methods such as molecular docking and quantitative structure–activity relationship (QSAR) models. Molecular docking predicts binding affinity by simulating the three—dimensional interaction of small molecules with target proteins. For example, docking has been used for virtual screening against TNF—α, a key inflammatory target in Uveitis [13]. The advantage of docking lies in providing interpretable details about molecular binding. However, its predictive accuracy is severely constrained by the precision of force field parameters and by treating protein structures as rigid, which limits the ability to simulate complex, flexible docking processes. Moreover, computational costs escalate dramatically as compound libraries grow.

QSAR models, on the other hand, correlate molecular descriptors with biological activity. Traditional machine learning models, for instance, have predicted anti—inflammatory activity using predefined molecular descriptors [14]. QSAR approaches offer computational efficiency, but their performance heavily depends on the quality of the descriptors. They struggle to capture deeper abstract features within molecular structures, resulting in limited generalization capabilities.

### 2.2. Screening model based on classical machine learning

To overcome these limitations, researchers have adopted more advanced classical machine learning algorithms such as random forests and support vector machines. These methods typically represent molecules using predefined fingerprints or descriptor vectors for classification or regression tasks. For instance, combining molecular fingerprints with a random forest algorithm enabled a classifier for anti—inflammatory agents that outperformed traditional QSAR models on public datasets [15]. The advantages of these methods include their simplicity, fast training times, and robustness with limited data. However, their performance is fundamentally constrained by manual feature engineering. Molecular fingerprints, being simplified representations, may lose critical structural information—such as precise topological relationships or the spatial orientation of functional groups—thus limiting predictive performance and hindering breakthroughs in complex tasks.

### 2.3. Modern graph neural network methods based on deep learning

In recent years, deep graph neural networks have become the new standard in AI—assisted drug discovery because they can directly process molecular graphs and automatically learn feature representations of atoms and bonds. Among these, graph convolutional networks (GCNs) and graph attention networks (GATs) have demonstrated particularly outstanding performance.

Graph Convolutional Network Models: GCNs have shown potential for directly handling graph—structured molecular data in property prediction. For example, applying GCNs to learn features from compound structures has been used to predict drug efficacy against retinal diseases [16]. GCNs are effective at aggregating information from local neighborhoods. However, their typical “message passing” mechanism treats all neighboring nodes equally, overlooking variations in the strength of atomic interactions. This can result in insufficient sensitivity to key functional groups.

Graph Attention Network Models: GATs introduce an attention mechanism to overcome GCN limitations, enabling models to dynamically assign different importance weights to neighboring nodes. GAT models have demonstrated superior performance across multiple molecular property prediction tasks. Compared to GCNs, GATs better capture key molecular substructures, and their predictions are often more interpretable [17]. However, the standard GAT attention mechanism suffers from computational rigidity: the attention scoring function between query and key vectors is fixed and may not adapt to varying graph contexts, leading to unstable performance in inferring complex molecular relationships.

### 2.4. Recent heterogeneous—network and attention—based methods in biomolecular prediction

In recent years, a class of models based on heterogeneous information network (HIN) and hierarchical attention mechanisms has shown significant effects in the field of prediction of drug—target interaction (DTI) and biomolecular associations. The HIN framework built by Su et al. improves DTI prediction performance by explicitly integrating multiple classes of nodes and multiple classes of edges such as drugs, proteins, phenotypes, etc [18]., utilizing network paths and semantic information (Su et al., 2022). The PPAEDTI proposed by Li et al. combines personalized propagation with autoencoders, spreading information on graph structures through propagation mechanisms and learning low—dimensional hidden representations [19], thereby enhancing robustness against sparse biological activity data (Li et al., 2022). On the other hand, the stratified attention + motif perception mechanism proposed by Zhao et al. demonstrates the value of local substructure (motif) information and stratified attention for capturing complex biological network relationships in miRNA—disease association prediction [20]. Similar work also includes a HIN learning method based on neighborhood—level structural representation, recently published in the Computational and Structural Biotechnology Journal, which further emphasizes the importance of neighborhood structure information and the complementarity of global heterogeneous relationships [21].

Although the above methods have obvious advantages in terms of entity and relationship integration at the network level, they are typically centered on entity/path—level semantics (e.g., drug—protein—phenotype triad relationships), while less directly model the fine—grained representations of atomic/key—level diagrams within molecules (i.e., molecular two—dimensional/three—dimensional diagrams) and perform deep fusion with traditional chemical descriptors. In contrast, the MLGT proposed in this paper focuses more on dynamic attention modeling of molecular diagrams (atom—level) and two—stream deep fusion with global chemical descriptors, thereby achieving fine—grained representations of drug activity discrimination while maintaining molecular microstructure information while balancing global drug chemical properties—this is complementary to HIN—like methods and is particularly suitable for applications that require precise capture of non—local interactions within molecules in conjunction with drug efficacy properties to determine activity.

### 2.5. Large language models and knowledge-graph–enhanced drug discovery

In addition to traditional molecular characterization learning, recent research has also explored the use of large language models (LLMs) as powerful feature extractors for drug discovery tasks [22]. By pre-training large-scale biomedical literature and structured entities, LLMs can encode rich semantic and relational knowledge that completes molecular structural information. Hybrid frameworks that combine LLM-generated embeddings with biomedical knowledge maps and graphic neural networks have achieved significant success in drug interaction prediction, drug-target interaction modeling, and drug safety analysis. These approaches highlight the value of incorporating external knowledge a priori into data-driven models, especially in situations with low data or high imbalances.

### 2.6. Multi-view and contrastive representation learning

Another closely related research direction is multi-view contrast learning, whose goal is to learn robust drug characterization by coordinating multiple complementary views through contrast targets. Typical views include molecular maps, chemical fingerprint maps, drug interaction networks, and biological annotations. By maximizing consistency between views while retaining discriminatory information, multi-view contrast methods have been shown to improve the generality and stability of drug-drug interaction event prediction and related pharmacological tasks [23]. These methods provide important methodological insights on how heterogeneous molecular information can be combined, providing the impetus for the multimodal fusion strategy adopted in this study.

### 2.7. Research status and gaps in drug screening for Uveitis

Despite the successes of GNNs in general drug discovery, their specialized application to Uveitis therapeutic screening remains in its infancy [24]. Current research primarily focuses on predicting generic anti—inflammatory or immunosuppressive activities, lacking dedicated models tailored to the multi—target and complex immunological context of Uveitis. First, publicly available Uveitis bioactivity data are limited and highly sparse, making data—driven deep learning models prone to overfitting. Second, many studies fail to effectively integrate auxiliary features such as molecular descriptors with graph structural information [25], thus not fully leveraging multi—source data. Finally, existing prediction models for Uveitis often directly apply general architectures without considering disease—specific pathobiology or customization (e.g., targeting specific pathways or biomarkers).

In summary, existing research has laid the groundwork for computer—aided Uveitis drug screening, but significant gaps remain. There is an urgent need for predictive models that deeply integrate molecular graph representations with multi—source features, incorporate efficient attention mechanisms, and are specifically optimized for Uveitis data. This study addresses these challenges by constructing an advanced GATv2—based framework, providing a powerful tool for the precise and efficient screening of anti—Uveitis lead compounds from vast chemical libraries. Table 1 in S1 File highlights the differences among various approaches.Table 2 in S1 File presents representative advanced artificial intelligence paradigms in recent drug discovery research and their relationship with machine learning generative technology (MLGT).

## Materials and methods

This study proposes MLGT (Multimodal Learning with Graph and molecular descriptors for Therapeutics. This nomenclature emphasizes the integration of graph—structured data (atoms and bonds) with continuous molecular descriptors—a multimodal approach that moves beyond conventional single—modality representations in molecular property prediction.), an end—to—end computational framework for virtual screening of Uveitis candidate drugs based on deep graph neural networks. The core model is a multimodal graph attention network that integrates molecular graph representations with chemical descriptor features. It employs a dynamic attention mechanism to capture internal molecular topology and bond interactions. Based on a compound’s SMILES string, the model outputs the probability of that compound inhibiting Uveitis—related biological targets. The overall workflow comprises four key modules: molecular graph construction, feature engineering, neural network architecture design, and training strategy.

### 3.1. Dataset

The data for this study were obtained from the ChEMBL database [26], a large manually curated repository of bioactive molecules. We identified key protein targets closely associated with the pathogenesis of Uveitis (e.g., TNF—α, IL—17, and components of the JAK—STAT pathway). We then used the ChEMBL Python API (chembl_webresource_client) to programmatically retrieve bioactivity data (including IC₅₀, Ki, Kd, etc.) of compounds targeting these proteins. Through the following rigorous filtering and standardization process, we finally constructed a carefully selected dataset containing approximately 300,000 high—confidence data points.

To ensure data consistency and reliability, we implemented a multi—step filtering and standardization process:

Activity Threshold Labeling [27]: Convert all activity data to IC₅₀ values. Compounds with IC₅₀ ≤ 10 μM are labeled as positive (active, label = 1), a threshold widely recognized in early drug discovery. Compounds with IC₅₀ > 30 μM are labeled as negative (inactive, label = 0). This stringent criterion ensures high confidence in negative samples. Compounds with IC₅₀ between 10–30 μM are excluded to reduce label noise.

Deduplication and Standardization: For molecules with multiple activity records, retain the most potent value. All molecules undergo SMILES string standardization using RDKit: conversion to canonical SMILES, removal of inorganic salts, and neutralization of charges.

Druggability Filtering: Apply Lipinski’s Rule of Five (150–800 Da molecular weight) and remove molecules containing reactive functional groups or undesirable structures using RDKit’s filter. This ensures the dataset focuses on drug—like chemical space.

To rigorously assess model generalization, we partitioned the data based on Bemis–Murcko scaffolds [28]. This scaffold—based splitting groups molecules by their core skeleton, ensuring that training, validation, and test sets contain distinct scaffolds. This strategy offers a more rigorous evaluation than random splitting, preventing models from overfitting to known structures and better simulating real—world drug discovery scenarios. Table 3 in S1 File shows the resulting dataset composition.

A sufficiently large test set is crucial for reliable performance estimation, especially in the high—dimensional chemical space characterized by enormous molecular diversity. A small test set may lead to high variance in metric estimations (such as AUC and F1 score) and fail to capture the long—tail distribution of biologically active compounds.To rigorously assess the model’s generalization ability, we implemented the Bemis–Murcko scaffold splitting method, ensuring that the training, validation, and test sets contain distinct molecular cores. This approach minimizes the risk of artificial performance inflation caused by structurally similar molecules appearing across splits. To further quantify the separation, we computed the maximum Tanimoto similarity (based on ECFP4 fingerprints) between any compound in the test set and the training set. The distribution of maximum similarities has a median of 0.35 and a 95th percentile of 0.55, confirming that the test set is predominantly composed of scaffold—novel molecules rather than close analogs of the training samples. This provides realistic simulation support for de novo virtual screening scenarios prioritizing novel chemotypes.

### 3.2. Experimental environment setup

The experiments were conducted on a Windows 11 workstation equipped with an Intel i7—10400 CPU, an NVIDIA GeForce RTX 5060 GPU, 256 GB of RAM, and Python 3.8.0. Table 4 in S1 File summarizes the experimental environment.

As shown in Table 5 in S1 File, we present the relevant model parameter data from the experiment.

### 3.3. Experimental methods

This study proposes an end—to—end deep learning framework for virtual drug screening based on graph neural networks. The core model is a Multi—modal Graph Attention Network, which integrates molecular graph representations with chemical descriptors. It employs a dynamic attention mechanism to capture the molecule’s internal topology and bond interactions. Given a compound’s SMILES string, the model outputs the probability of it exhibiting anti—Uveitis activity. The overall workflow comprises four modules: molecular graph construction, feature engineering, neural network architecture design, and training strategy.

In representing molecules, each compound is depicted as a graph where atoms serve as nodes and chemical bonds as edges. We encode a comprehensive set of atomic features: atom type (one—hot encoding for 9 common elements), degree (normalized), formal charge (normalized), chirality, number of bonded hydrogens, hybridization, aromaticity, atomic mass (normalized), ring membership, and ring size (3–8 members). Bond features include bond type (one—hot encoding for single, double, triple, aromatic), conjugation, ring involvement, and stereochemistry. All molecules are preprocessed with RDKit: invalid SMILES are removed, SMILES strings are canonicalized, salts are removed, and charges are neutralized to ensure data quality. Additionally, we compute 50—dimensional global molecular descriptors (e.g., molecular weight, LogP, hydrogen bond donors/acceptors, topological polar surface area, rotatable bond count, and ring counts) to complement the graph representation with rich physicochemical information.

The core of our architecture is the GATv2 layer, which introduces a dynamic attention mechanism that addresses the limitations of the standard GAT. In the standard GAT, the calculation of the attention coefficient α_ij_ between nodes **i** and **j** is as follows:


$$\alpha_{\text{ij}}=\text{exp}(\text{LeakyReLU}({\text{a}}^{⊤}[\text{W}{\text{h}}_{\text{i}}||\text{W}{\text{h}}_{\text{j}}]))/\sum \text{k}\in \text{N}(\text{i})\text{exp}(\text{LeakyReLU}\left({\text{a}}^{\text{T}}\left[\text{W}{\text{h}}_{\text{i}}||\text{W}{\text{h}}_{\text{k}}]\right)\right),$$

where h_i_ is the characteristic vector of node **i**, **W** is the learnable weight matrix, **a** is the learnable attention vector, and ∥ represents the connection. This representation is static because the attention score function is fixed and applies uniformly across all node pairs, possibly lacking adaptability to different graph contexts.

In contrast, GATv2 modifies the order of operations to enable dynamic attention:


$$\alpha_{\text{ij}}=\text{exp}({\text{a}}^{⊤}\text{LeakyReLU}(\text{W}[{\text{h}}_{\text{i}}||{\text{h}}_{\text{j}}]))/\sum \text{k}\in \text{N}(\text{i})\text{exp}({\text{a}}^{\text{T}}\text{LeakyReLU}(\text{W}[{\text{h}}_{\text{i}}||{\text{h}}_{\text{K}}]))$$

Here, a nonlinear activation function (LeakyReLU) is applied to attention computation, allowing the attention mechanism to dynamically adjust its importance based on the combined characteristics of neighboring nodes. This design enables the model to capture context—dependent interactions and assign adaptive weights to different atomic neighbors, which is crucial for modeling complex molecular diagrams.

Why is dynamic attention particularly suitable for capturing non—local atomic interactions in molecules?

Variable interaction intensities: In molecular maps, atomic interactions are not uniformly distributed; certain functional groups or pharmacological groups may have stronger effects on biological activity. Dynamic attention in GATv2 can adaptively balance these interactions, enhancing sensitivity to key substructures.

Long—range dependencies: Non—local interactions (such as hydrogen bonds, electrostatic effects) typically occur between atoms that are far apart in the diagram but are critical for binding. Dynamic mechanisms enable the model to propagate signals beyond the immediate neighboring atoms, effectively capturing this dependency.

Chemical environmental adaptability: Molecules exhibit different electronic and stereoscopic environments. Dynamic attention mechanisms can adjust their scoring functions according to the local chemical environment, providing more flexible and expressive representations compared to static attention.

This theoretical advance supports the ability of this study model to outperform standard GAT and other GNN variants in predicting staphylococcus—related biological activity, which was confirmed in the study of the dissolution of this study.

Our predictive model uses the Graph Attention Network v2 (GATv2) architecture, which is well—suited for capturing complex non—local interactions between atoms via its dynamic attention mechanism. The model consists of three GATv2 layers, each with eight attention heads. The first layer projects the 78—dimensional atomic features into a 256—dimensional hidden space, followed by batch normalization and an ELU activation. The subsequent layers maintain the 256—dimensional hidden space and iteratively refine node embeddings through message passing. Each GATv2 layer (except the last) uses multi—head outputs to preserve model expressiveness and applies a dropout of 0.2 to prevent overfitting. The final GATv2 layer outputs 256—dimensional atom—level embeddings.

We aggregate the atom—level embeddings into a graph—level representation using a dual pooling strategy: combining global average pooling and global max pooling. This yields a 512—dimensional vector that captures both average structural properties and the most salient atomic features. We then fuse these graph embeddings with the molecular descriptors. The descriptors are projected through a two—layer fully connected network with ReLU activations (reducing to 64 and then 32 dimensions) and dropout, and concatenated with the graph embedding to form a 544—dimensional feature vector. This fused vector is fed into a two—layer feedforward network (FFN) with 512 hidden units, batch normalization, ReLU activation, and dropout, culminating in a single output neuron that predicts the probability of therapeutic activity.

During training, we employ robust techniques to address class imbalance and label noise. We use a binary cross—entropy loss with label smoothing (ε = 0.1), which relaxes the hard targets (0/1) to soft labels (0.05/0.95). This reduces overfitting and improves calibration. Class weights are computed, and a weighted random sampler is applied during mini—batch sampling to enforce an expected 1:1 ratio of positive to negative samples, preventing the majority class from dominating the gradient. We also use gradient accumulation with a step size of 2 and gradient clipping at 3.0, which simulates a larger effective batch size (128) while using batch size 64 (limited by GPU memory), improving training stability. The learning rate follows a Cosine Annealing with Warm Restarts schedule (T₀ = 10, T_mult = 2, η_min = 1e—7), helping the optimizer escape sharp local minima. We apply early stopping based on validation AUC: if AUC does not improve by at least 0.001 over 20 epochs, training halts and the model reverts to the best recorded weights, further preventing overfitting.

Additionally, we apply SMILES randomization for data augmentation. Each molecule is represented by multiple randomly permuted SMILES strings; with an augmentation ratio of 0.3, we generate up to three different SMILES variants per molecule. This enhances the model’s robustness to input variations and encourages it to learn representations invariant to SMILES syntax.

We evaluate performance using multiple metrics: AUC—ROC (primary metric), accuracy, precision, recall, and F1 score, ensuring a comprehensive assessment. All experiments are implemented in PyTorch Geometric and leverage NVIDIA GPUs (DataParallel). A fixed random seed of 42 ensures reproducibility. Fig 1 in S1 File illustrates our overall model architecture.

The analysis and comparison of the GATv2 model architecture are shown in Figs 2 and 3 in S1 File, respectively

Fig 2 in S1 File intuitively compares the key differences between the dynamic attention mechanism of GATv2 and the static mechanism of the standard Graph Attention Network (GAT) in molecular graph representation learning: GATv2 assigns variable weights to atom interactions in molecular graphs through its dynamic, context—adaptive attention function (e.g., via adaptive computation using LeakyGLU activation), enabling it to flexibly respond to subtle changes in different chemical environments and thereby accurately capture the nonlinear and long—range atomic interactions crucial for biological activity; in contrast, the static attention mechanism adopted by traditional GAT has a fixed computational pattern, with relatively rigid weight assignment that is decoupled from specific topological contexts, making it difficult to fully model the dynamic and differentiated correlation strengths between functional groups in complex molecules. This schematic further illustrates core concepts in molecular graphs (e.g., central atoms, neighbor atoms, and attention weights) and emphasizes that the dynamic adjustment capability of GATv2 allows it to identify and enhance the contribution of key pharmacophoric features (e.g., specific functional groups), which provides a more reliable computational foundation for the precise screening of lead compounds from structurally complex compound libraries.

Fig 3 in S1 File presents the end—to—end architecture of the GATv2—enhanced multimodal neural network (MLGT) proposed in this study. This framework achieves precise virtual screening by synergistically integrating the topological structure of molecular graphs (78—dimensional atomic properties and 12—dimensional bond properties) with global physicochemical descriptors (50—dimensional). The core of the architecture consists of five GATv2 graph attention modules, which employ a dynamic attention mechanism that enables attention weights to be adaptively computed based on graph context. These modules also leverage eight attention heads in parallel to capture diverse chemical interaction patterns. Each layer is 辅以 with batch normalization, ReLU activation, dropout, and residual connections to ensure training stability and discriminative representation. Following hierarchical graph representation learning, atomic—level features are aggregated into graph—level representations via a dual pooling strategy (global average and max pooling). These representations are then deeply fused with molecular descriptors refined by a feedforward network, forming a 544—dimensional unified multimodal feature vector. The final probability prediction of the compound’s anti—uveitis activity is output by a classifier. This design realizes an automated, high—precision mapping from raw molecular information to biological activity prediction, embodying the organic integration of dynamic attention, multi—source feature fusion, and advanced regularization strategies. It provides a powerful and interpretable deep learning paradigm for computational drug discovery targeting complex disease targets.

### 3.4. Evaluation model

To comprehensively evaluate our GATv2 model for virtual screening, we employ a multi—level assessment framework. This evaluation not only measures the model’s overall discriminative capability but also examines its robustness, generalization ability, and the contributions of its key components.

We also conducted a preliminary analysis of prediction uncertainty. By computing the entropy of the model’s output probabilities on the test set, we identify cases where the model is “uncertain” (high entropy) [29]. These molecules are often structurally novel or lie near decision boundaries, and their predictions should be prioritized for experimental validation. Quantifying uncertainty thus paves the way for active learning, enabling iterative model refinement.

Despite the model’s complexity, its computational efficiency enables large—scale screening. On a single server with two NVIDIA RTX 5060 GPUs, a forward pass on 300,000 compounds takes only about five minutes (~1 millisecond per molecule). This demonstrates the significant speed and cost advantages of AI—driven methods over traditional high—throughput screening.

### 3.5. Implementation details for baseline models

To ensure fairness, reproducibility, and rigorous comparison, all baseline models were implemented using PyTorch Geometry 2.0 and scikit—learn 1.2, trained on the same scaffold—split dataset, and evaluated using the same metrics. Hyperparameters for each baseline were optimized via grid search on the validation set to report their potential best performance. Common training settings for all neural baselines included: AdamW optimizer, batch size of 128, cosine annealing with warm restarts learning rate scheduler (T0 = 10, ηmin = 1e—7), gradient clipping of 3.0, and early stopping after 20 epochs based on validation AUC. Specific configurations for each baseline are as follows:

GCN: Three graph convolution layers with 256 hidden channels, batch normalization, ReLU activation, and a dropout rate of 0.2. Graph—level representations were obtained via global average pooling, followed by a two—layer feedforward classifier (512 hidden units).

GAT (Standard): Three graph attention layers with 8 attention heads per layer and 256 hidden channels per head. The concat operation was applied to the multi—head outputs. Dropout (0.2) was applied to both attention weights and node features. This model used only graph inputs (atoms and bonds) without molecular descriptors.

Attention FP: We used the architecture recommended by the authors, with a node—level attention depth of 3 and a graph—level attention depth of 3. The hidden size was set to 256, and a dropout of 0.2 was applied. The initial learning rate was set to 1e—3.

Chemprop: We used the official implementation with default MPNN parameters: hidden size of 300, depth of 3 (message passing steps), and 2 feedforward layers in the final predictor. The dropout rate was 0.2.

RF—SMILES: Molecules were encoded into 2048—bit ECFP6 (radius = 3) fingerprints using RDKit. A random forest classifier from scikit—learn was trained with 500 estimators (n_estimators = 500), no maximum depth limit, and with class_weight = ‘balanced’ to reduce label imbalance.

These detailed implementations ensure the reliability of comparability, and the advantages reported for MLGT should be attributed to its architectural innovations rather than suboptimal benchmarking.

## Results

### 4.1. Experimental results

We evaluated the anti—Uveitis activity prediction framework of the hybrid model architecture MLGT, assessing its performance across three dimensions: accuracy, generalization capability, and computational efficiency.

As shown in Figs 4 to 6 in S1 File, the model demonstrated outstanding performance on the ChEMBL test set: achieving 97.7% accuracy, an F1 score of 97.2%, and a recall rate of 96.1%. The learning curve demonstrates rapid improvement and convergence within 51 training epochs. All metrics steadily increased from initial values (approximately 0.80) to final values (approximately 0.96–0.97), achieving a relative improvement of 20%. This trend indicates efficient optimization via gradient descent. Notably, the earliest training phase (epochs 1–20) exhibits the fastest improvement rate, consistent with larger gradient changes during initial training. In the later phase (epochs 30–50), metrics stabilize with minor fluctuations, indicating the model approaches convergence.

Overall, the model exhibits excellent training dynamics and convergence properties. The simultaneous improvement in accuracy, F1 score, and recall demonstrates its robust feature learning and classification capabilities. Minor fluctuations during training are normal and can be further reduced through hyperparameter tuning. The final model achieves outstanding performance on the test set: accuracy > 0.97, F1 score > 0.97, recall > 0.96.

In addition, the high accuracy (97.7%), F1—score (97.2%), and recall (96.1%) observed in this study reflect the model’s strong discriminative power on the curated test set. We attribute this performance to several synergistic factors:

1. Multimodal Feature Integration: The fusion of graph—based atomic representations with global physicochemical descriptors captures both local and global determinants of bioactivity.
2. Advanced Regularization: Label smoothing, class—balanced sampling, and gradient accumulation collectively mitigate overfitting and improve model calibration.
3. High—Quality Dataset Curation: The stringent filtering pipeline ensures that both active and inactive labels are of high confidence, reducing noise that typically plagues bioactivity datasets.

Nevertheless, we acknowledge that while these metrics are impressive, they must be interpreted in context: the test set, though scaffold—separated, remains within the broad chemical space defined by Uveitis—related targets. Performance on entirely out—of—distribution chemotypes or alternative assay formats may vary, highlighting the importance of continued external validation.

### 4.2. Receiver operating characteristic

The Receiver Operating Characteristic (ROC) curve is a vital tool [30] for evaluating binary classification models, as it visualizes the trade—off between True Positive Rate (TPR) and False Positive Rate (FPR) across thresholds. Fig 7 in S1 File shows the ROC curve for our model. The orange curve represents the trained model’s performance, with an AUC of 0.9156, indicating excellent discrimination between active and inactive compounds. In the low—FPR region (e.g., FPR < 0.2), the TPR rises rapidly—at FPR = 0.2, TPR is already quite high—signifying that the model has high sensitivity and captures a large fraction of positive cases early while controlling false positives. The blue dashed diagonal line is the random baseline (AUC = 0.5); the actual curve’s significant upward deviation from this baseline underscores the model’s predictive advantage.

Orange curve (actual ROC curve): Represents the performance of the trained model, with an AUC value of 0.9156. This indicates a large area under the curve, signifying the model’s excellent performance in distinguishing positive and negative examples. The curve shape shows that in the low FPR region (e.g., FPR < 0.2), TPR rises rapidly. For instance, when FPR = 0.2, TPR may reach a relatively high value. This trend indicates the model possesses high sensitivity, capable of capturing a large number of positive cases at early thresholds while controlling false positives.

Blue dashed line (random curve): Represents the baseline for random guessing, with AUC = 0.5. This diagonal line indicates equal TPR and FPR (meaning the model has no discriminative power, equivalent to flipping a coin). In contrast, the actual curve shifts significantly upward and to the left, highlighting the model’s predictive advantage.

Overall, the ROC analysis demonstrates the model’s outstanding performance in this binary classification task. With an AUC of 0.9156, the model far exceeds random chance. This visualization not only confirms the model’s discriminative capability but also provides guidance for threshold selection. Future work may focus on validating generalization on independent datasets and analyzing feature importance to further enhance performance.

### 4.3. Comparative experiment

To rigorously evaluate the effectiveness of our proposed MLGT framework for Uveitis drug screening, we designed a comprehensive set of comparative experiments. Table 6 in S1 File summarizes the selected benchmark models, representing a spectrum of approaches ranging from classical machine learning to state—of—the—art graph learning techniques.

The first experiment employed a GCN (Graph Convolutional Network) approach using Kipf & Welling’s graph convolutional architecture, updating node representations through mean aggregation of neighboring nodes. A basic GNN model was constructed with three layers of GCN followed by global pooling and a fully connected classifier, demonstrating the necessity of attention mechanisms (rather than simple neighborhood aggregation) in molecular representation learning. GATv2 was expected to significantly outperform GCN due to its dynamic attention weight allocation capability, achieving 4.6% higher accuracy than our architecture.

To isolate the contribution of the dynamic attention mechanism in GATv2, we implemented a standard GAT baseline using the same hyperparameter configuration as our GATv2 layers (3 layers, 8 attention heads, 256 hidden channels) but without the dynamic attention formulation. This model processes only the molecular graph without incorporating molecular descriptors. The results show that GAT outperforms GCN by +1.7% in accuracy, demonstrating the advantage of attention over mean aggregation. However, it still underperforms our full MLGT model by —2.9% in accuracy, highlighting the synergistic gain from both GATv2’s dynamic attention and multimodal descriptor fusion.

The third experiment employed Attentive FP (Attention Fingerprint Model) [31], utilizing an attention—based message passing mechanism that incorporates both node—level attention and global graph—level attention pooling. This evaluated the impact of different attention architectures (message passing attention vs. GATv2’s node—pair attention) on molecular property prediction. Accuracy decreased by 6.1% compared to our architecture.

The fourth experiment employed Chemprop (Directed Message Passing Network) [32], implementing a D—MPNN architecture that avoids over—smoothing by updating edge (rather than node) features. It compared performance differences between message passing paradigms (node—centric vs. edge—centric). Accuracy decreased by 8.0% compared to our architecture.

The fifth experiment employed RF—SMILES (Random Forest—SMILES), generating 2048—bit ECFP6 molecular fingerprints using RDKit. These fingerprints were input into a random forest classifier (n_estimators = 500) as a representative traditional virtual screening method, validating the advantages of deep learning models over conventional machine learning approaches. This method showed a 3.5% reduction in accuracy compared to our architecture.

### 4.3. Ablation study

To systematically evaluate the contributions of each key component in the proposed GATv2 model (Proposed Model), we designed and conducted comprehensive ablation study. All experiments were performed under the same training/validation/test dataset partitioning to ensure comparability of results. Evaluation metrics included accuracy, F1—score, and recall. The experimental results are shown in Table 7 in S1 File.

In the first experiment, we removed the custom attention pooling layer from this variant model and replaced the graph pooling strategy with the original global mean pooling. All other components and hyperparameters remained consistent with the full model. Compared to the full model, this variant exhibited a decrease in accuracy of approximately 4.9 percentage points, along with a noticeable decline in recall. This indicates that simple mean pooling may lose information from certain critical atomic nodes within molecular graphs. In contrast, the attention mechanism dynamically learns and weights the importance of different atomic nodes for the final prediction task. It more effectively captures molecular substructures relevant to Uveitis drug activity, thereby enhancing the model’s ability to perceive key features and improve its generalization performance.

In the second experiment, we removed the 50—dimensional molecular descriptor features computed from RDKit in this variant model. The model made predictions solely based on representations learned from the molecular graph structure via GATv2 to validate the effectiveness of multimodal feature fusion. This variant exhibited the most significant performance decline (F1 score decreased by 7.5 percentage points), strongly demonstrating that global molecular descriptors—such as molecular weight, LogP, TPSA, etc.—provide crucial global chemical information that the graph structure alone struggles to capture directly. These descriptors are closely correlated with drug pharmacokinetic properties (e.g., membrane permeability, solubility). Their inclusion provides complementary information to the model, proving essential for distinguishing promising drug candidates.

To explicitly evaluate the contribution of GATv2’s dynamic attention mechanism, we replaced the GATv2 layers in MLGT with standard GAT layers while keeping all other components (including molecular descriptors) unchanged. This variant achieved 94.8% accuracy, which is 2.9% lower than MLGT. This indicates that the dynamic attention mechanism in GATv2—which adaptively computes attention weights based on both query and key vectors—provides a measurable performance uplift over static GAT attention, particularly in capturing non—local atomic interactions relevant to Uveitis bioactivity.

In the forth experiment, we removed the WeightedRandomSampler from this variant model and trained it using a standard random sampler. This experiment was conducted to validate the necessity of category balancing strategies on typical imbalanced datasets like drug screening. After removing category balancing, the model’s recognition capability for the minority class (active molecules) declined sharply, with the Recall value dropping significantly by approximately 7.2 percentage points. This indicates the model tends to correctly predict the majority class (inactive molecules) while neglecting learning about active molecules. The category balancing strategy, by resampling, forces the model to treat positive and negative samples equally, greatly improving its ability to discover scarce active compounds. This holds significant practical importance for high—throughput virtual screening applications.

In the fifth experiment with this variant model, we replaced the loss function with standard binary cross—entropy loss (BCEWithLogitsLoss) [33]and removed the label smoothing regularization technique. The accuracy and F1 score of this variant remained lower than those of the full model. Label smoothing mitigates model overfitting to training samples by softening hard labels, thereby enhancing calibration and generalization capabilities. This effect proves particularly valuable with limited bioactivity data.

### 4.4. Feature importance and redundancy analysis

To further investigate how different atomic and key—level features affect the predictive ability of the proposed model, we conducted a comprehensive feature importance and redundancy analysis.

Intergroup dissolution results. When the atom features associated with aromaticity and hybridization were removed, the model exhibited the most significant performance degradation, with an average decrease of about 0.018 percentage points in AUC and a decrease of 2.0 percentage points in the F1 score. This observation highlights the crucial role of aromaticity and hybridized substructures in distinguishing active and inactive compounds. In contrast, the removal of certain geometrically related features only resulted in marginal performance changes, indicating the presence of partial redundancy.

Key Characteristic Contribution. The elimination of key types and conjugate characteristics resulted in a sustained decline in prediction accuracy, suggesting that explicitly encoding key sequences and electron deslocation are crucial for capturing structural patterns that are chemically significant. Arrangement importance analysis further confirms that conjugate and aromatic bonds are one of the main contributors to model performance.

Attention—based and attribution—based analysis. The analysis of GATv2 attention weights showed that the atoms that obtained high attention scores were mainly heterotomes (e.g., N and O) and aromatic carbon, especially in compounds predicted to be active. This pattern is consistent with known drug—target interaction mechanisms, where heterotomes are usually involved in hydrogen bonding, and aromatic parts contribute to hydrophobic and π—π interactions. SHAP analysis of global descriptive branches showed that features associated with lipophilia and polarity had the greatest impact on prediction results.

Redundancy assessment. Correlation and VIF analysis identified several highly correlated dimensions between atomic mass and atomic type—related features. The performance loss caused by removing these dimensions is negligible. This indicates that some low—level features may be redundant when advanced chemical information has already been encoded.

Summary. Overall, these results show that while most of the selected atomic and bond characteristics are informatively rich and necessary, a small subset exhibits redundancy. More importantly, pharmacological and chemical—based characteristics—such as aromaticness, heteratomic presence, and bond conjugation—always contribute the most to model performance, thereby improving prediction accuracy and interpretability.

### 4.5. Computational complexity and resource consumption analysis

Although predictive performance is crucial for virtual screening, the practical suitability of models in large—scale drug discovery scenarios also depends on their computational complexity and resource requirements. In this section, we conduct a systematic analysis of the model size, memory consumption, and computational efficiency of the mentioned MLGT framework during the training and inference stages.

The MLGT model proposed in this study consists of a three—layer GATv2 trunk with multihead attention, a descriptor processing branch, and a lightweight feedforward classifier head. Under the configuration described in Table 4 in S1 File, the total number of trainable parameters in the MLGT is approximately 2.8 million, with the GATv2 layer occupying the majority of the parameters due to multihead attention projection, while the descriptor branch and final classifier contribute to the marginal overhead. Compared to the standard GAT and Chemprop baselines, under comparable hidden dimensions, the MLGT introduces only moderate increases in the number of parameters, while providing significantly improved predictive performance, indicating that the performance improvement is not achieved through excessive parameter scaling.

In the experimental setting described in Section 3.2, we measured GPU memory usage on NVIDIA RTX 5060 GPUs. During training, with an effective batch size of 128 (realized through gradient accumulation), MLGT exhibited peak GPU memory consumption of approximately 3.8 GB, which still maintained well within the capacity range of a single consumer—grade GPU. During inference, memory usage decreased significantly due to no longer needing gradient storage and optimizer states. In this setting, peak GPU memory usage decreased to approximately 2.3 GB, enabling efficient deployment of large—scale virtual selection tasks.

To evaluate inference efficiency, we benchmarked the end—to—end positive transmission of MLGT on a dataset containing about 300,000 compounds. In a dual GPU setting (using two RTX 5060 GPUs with data parallelization), the complete virtual filtering process was completed in about 5 minutes, with corresponding average inference time of about 1 millisecond per molecule. This throughput indicates that MLGT can scale linearly with dataset size, suitable for high—throughput virtual filtering scenarios involving hundreds of thousands to millions of candidate compounds.

From a theoretical perspective, the main computational cost of MLGT stems from GATv2 message passing operations. For N atoms and E bonds, each GATv2 layer has a sequentially arranged temporal complexity O(E●H), where H represents the hidden feature dimension. The use of multi—head attention increases the constant factor, but does not change the asymptotic complexity. Importantly, molecular graphs are typically smaller and sparse (with a limited number of atoms and bonds), which in practice maintains a lower absolute computational cost. The descriptor processing branch consists of simple fully connected layers, and the additional computational overhead is negligible compared to the graph attention backbone.

Overall, this analysis shows that MLGT achieved a favorable balance between computational efficiency and predictive accuracy. Although the incorporation of dynamic attention and multimodal fusion slightly increased model complexity compared to simpler graph—based baselines, the resulting resource requirements remained moderate and maintained good consistency with actual hardware constraints. Crucially, significant improvements were achieved in predictive performance and robustness, and there was no significant increase in memory usage or inference time, making MLGT a computationally efficient and practically deployable large—scale anti—stagococcal drug screening solution. Table 8 in S1 File presents the complexity analysis of the MLGT model

### 4.6. Uncertainty analysis and its implications for experimental prioritization

To quantify the confidence of the model in its predictions and identify the compounds that require further experimental validation, we performed an uncertainty analysis based on the entropy of the output probability distribution for each test sample.

Calculate H(p) = —plogp—(1—p) log(1—p), where p represents the predicted probability of anti—glycinitis activity.

As shown in Fig 8 in S1 File, in a test set of 12,500 samples, the model was able to clearly identify 10.0% (1,250) of the “high uncertainty” predictions through the entropy value (threshold of 0.986). The accuracy of this portion of the predictions (mean 0.819) was significantly lower than that of the “low uncertainty” sample (mean 0.974), indicating that the model‘s uncertainty estimation is an effective proxy indicator of prediction reliability.

The framework constructed by this study, MLGT, implements a full—chain analysis from the uncertainty distribution, the relationship with confidence, to the final correlation of prediction errors. It not only warns of unreliable predictions, but can also diagnose their root cause (through dimensions such as structural novelty, probability distribution, etc.). This marks the shift from pursuing a single accuracy metric to providing a complete report of “predictive results and their confidence level”, which is a critical step for computational models to reach robust practical applications. This work demonstrates that an AI model with the ability to quantify uncertainty has value not only in making predictions, but also in identifying the limitations of its own knowledge. Isolating about 10% of high—uncertainty predictions and assigning them to experimental priority validation can greatly optimize the allocation of R&D resources. This establishes a new set of reliability standards and validation paradigm for AI—driven drug discovery, namely, “trusted predictions must include self—awareness of their uncertainty,” paving the way for driving the field from “black box” predictions to “transparent, auditable, high—stability” decision support systems.

We further investigated the relationship between prediction uncertainty and model performance. As shown in Table 7 in S1 File, the prediction accuracy rate for low entropy (H < 0.3) was 98.5%, and the F1 score was 98.1%, while the accuracy rate (76.2%) and the F1 score (74.8%) for high uncertainty prediction (H ≥ 0.6) were significantly lower. This highlights the utility of entropy as a reliable indicator of prediction reliability. In actual screening scenarios, high uncertainty compounds can be labeled for prioritized experimental validation, thereby implementing an active learning workflow that iterates with targeted new data to improve the model.Table 9 in S1 File shows the performance of the model stratified by prediction uncertainty.

## Module

### 5.1. Data preprocessing and molecular graph representation

This module converts molecular SMILES strings into graph structures and extracts atom and bond features for the GNN input. Each atom is represented by a 78—dimensional vector containing: atom type (9—dimensional one—hot encoding), normalized degree, normalized formal charge, chirality, hydrogen count, hybridization, aromaticity, atomic mass, and ring information (6 dimensions for ring size). Each bond is represented by a 12—dimensional vector: bond type (4—dimensional one—hot encoding for single, double, triple, aromatic), conjugation indicator, ring indicator, and stereochemistry. Additionally, we compute a 50—dimensional vector of global physicochemical descriptors (e.g., molecular weight, LogP, TPSA) which is fused with the graph features. By embedding this structured chemical knowledge into the input layer, the model gains a rich representation of molecular topology and electronic properties.

### 5.2. GATv2—based Graph Neural Network

This study employs an enhanced Graph Attention mechanism (GATv2) for hierarchical representation learning on molecular graphs. The model architecture consists of three GATv2 convolutional layers, each producing 256—dimensional node features, with eight attention heads per layer (yielding 2048—dimensional features when concatenated). We apply batch normalization and ELU activations between layers, and we concatenate multi—head outputs for expressiveness. We also support edge attribute injection to propagate bond—level information alongside node features.

### 5.3. Global map pooling and multimodal fusion module

This module performs two critical functions: (1) aggregating the refined, context—aware atom embeddings from the GATv2 layers into a single vector representing the entire molecule, and (2) fusing this structural representation with supplementary physicochemical descriptors.

Graph—Level Readout via Dual Pooling: The dynamic attention mechanisms in GATv2 have already assigned importance to atoms within their local and global contexts. To synthesize this information at the graph level, we adopt a dual—pooling readout operation. Formally, given the final atom embeddings {hi}^N^i = 1, the graph embedding g is computed as:g=[GAP({hi})||GMP({hi})], where GAP denotes global average pooling, GMP denotes global max pooling, and ∥ is the concatenation operator. This approach provides a balanced and efficient summary: GAP offers a stable estimate of the general molecular feature distribution, while GMP acts as a detector for the most salient atomic states, which are often indicative of bioactive moieties. We intentionally avoid introducing an additional learned attention layer at this stage to maintain model simplicity and prevent overfitting, relying instead on the powerful feature discrimination already achieved by the preceding GATv2 layers.

Descriptor Processing and Fusion: Concurrently, the set of 50—dimensional molecular descriptors is processed through a two—layer fully connected network with ReLU activations and dropout. This non—linear transformation projects the descriptors into a latent space that is dimensionally aligned and semantically enriched. The processed descriptor vector d is then concatenated with the graph embedding g to form the unified multimodal representation z=[g||d]. This fused vector z comprehensively encodes both the topological nuances learned by the GNN and the global chemical properties, serving as the input to the final classifier.

### 5.4. Theoretical basis and contribution analysis of atom and bond features

Although the atomic and bond—level features adopted in this study follow widely used molecular diagram representations, it is crucial to clarify their theoretical basis and quantitatively evaluate their practical contribution to predictive performance. The feature selection in this study was guided by established pharmacochemical principles and known pharmacological mechanisms of drug—related meningitis.

Theoretical motivation. The selected atomic characteristics encode physicochemical properties closely related to drug—target interactions and pharmacokinetics behaviors. Specifically, atomic types, heterozygosity, and aromaticity describe the local electronic environment and orbital configuration, which are crucial for π—π accumulation, hydrophobic interactions, and coordination with protein residues. Hydrogen count and positive charge are directly related to hydrogen bond supply/receptor capacity and electrostatic interactions, which play crucial roles in molecular identification and binding affinity. Ring members and ring size capture structural rigidity and flatness, which affect binding specificity and membrane transparency. Similarly, bond characteristics such as bond sequence, conjugation, and stereochemical characterization of electron de—location, molecular flexibility, and three—dimensional geometry, all of which are known to affect ligand stability and biological activity.

Quantitative contribution analysis. To go beyond qualitative proof, we systematically evaluated the importance and necessity of individual atomic and key features through a unified contribution analysis framework. First, intergroup and single feature dissolution experiments were conducted by selectively removing predefined feature subsets (such as aromatic—related atomic features or conjugate—related key features) and measuring the resulting changes in AUC, F1 scores, and recall rates. Second, an arrangement importance analysis was applied to the test set, where each feature dimension was randomly arranged while keeping the other feature dimensions unchanged, allowing us to estimate the information contribution of each feature to model performance.

Model—based interpretability. Furthermore, the model‘s intrinsic interpretability was investigated using attention—based statistics and gradient—based attribution methods. For the GATv2 layer, atomic—level attention weights were aggregated between the head and layer, and the attention score distributions between active and inactive compounds were compared. This analysis enabled the identification of atomic types and key patterns that consistently received higher attention during the prediction process. For the global descriptor branches, SHAP—based interpretation was adopted to quantify the contribution of individual physicochemical descriptors to the final output.

Redundancy and noise evaluation. To check for potential redundancy between features, correlation analysis and variance inflation factor (VIF) diagnosis were performed on the input feature matrix. Highly correlated or co—linear dimensions were further evaluated by control removal to assess whether their exclusion leads to performance degradation.

Pharmacological—driven feature expansion. Finally, considering the inflammatory and immune—regulating mechanisms behind grapevine inflammation, we explored several domains—informed candidate features, including the functional group count common in anti—inflammatory small molecules (such as amines, sulfonamines, and aromatic heterocyclics), as well as acidity/alcalinity—related indicators. These features were gradually integrated and evaluated to examine whether pharmacological knowledge—driven feature design could further improve model performance and interpretability.

All contribution analyses were repeated multiple times with different random seeds, and statistical significance was evaluated using appropriate tests (such as the DeLong test for AUC) to ensure the robustness and reliability of the reported results.

## Discussion

### 6.1. Addressing core challenges in AI—driven drug screening for Uveitis

Despite the widespread use of high—throughput screening (HTS) and virtual screening (VS), targeted drug discovery for complex diseases such as Staphylococcus pneumonia remains hampered by basic computational challenges. This study not only identifies these challenges, but addresses them directly through the design and validation of a multimodal MLGT framework, as our most advanced research results demonstrate. Below, we place our findings in the context of the main obstacles to show how MLGT provides specific solutions.

First, regarding the scarcity and noise of high—quality labeled data, which severely limits the generalization of the model, our method provides multiple mitigation strategies. MLGT‘s excellent performance (accuracy: 97.7%, AUC—ROC: 0.9156) on rigorously screened scaffold segmentation datasets directly demonstrates its robustness against overfitting—a common consequence of data scarcity. This robustness is not accidental, but was achieved through several key innovations: (1) Advanced regularization: We achieved label smoothing (ε = 0.1) and class balance sampling, which significantly reduced the model‘s overconfidence in potential noise labels, and enforced fair learning from a small number of (active) classes. Dissolving studies (Table 6 in S1 File) confirmed their key role, showing that F1 scores decreased by about 8% in the absence of class balance. (2) Increase data through SMILES randomization: This technique effectively extends the perceptual diversity of our training set, without the need for additional experimental data, thereby teaching the immutable model representation of molecular structures. (3) Multimodal feature fusion: By combining graph—based features with complementary molecular descriptors, MLGT reduces dependence on any single, potentially noisy data mode. In our dissolution study, the most significant performance decline occurred when molecular descriptors were removed, emphasizing that this fusion provided more stable and informative learning signals, buffering defects in graph—only data. Therefore, the performance of MLGT directly demonstrates that a combination of algorithm regularization, synthetic data expansion, and robust feature integration can successfully overcome the “scarcity and noise” problem in Uveitis biological activity data.

Second, in the trade—off between molecular representation integrity and computational efficiency, the dual—stream architecture of the MLGT provides a principled compromise scheme. We do not rely solely on two—dimensional graphics (lack of global properties) or static descriptors (loss of topological nuance), but combine both synergistically. The GATv2 main trunk efficiently captures complex atomic interactions and key properties, while the parallel descriptor branches integrate key physicochemical spectra (e.g., LogP, TPSA). This design reflects the dual considerations of local interactions and global drug similarity among pharmacologists. Crucially, this multimodal integration was achieved with high computational efficiency – 300,000 compounds were screened out in about 5 minutes. This shows that it is possible to transcend the limitations of single—modal representation without incurring daunting computational costs, effectively balancing information integrity with practical utility.

Third, regarding insufficient explicability and limited generalization, the framework of this study makes deliberate progress. Although complete explicability remains a field—wide challenge, MLGT uses the dynamic attention mechanism of GATv2 to provide fundamental tools for future explanatory analysis. Unlike static GAT or pooling methods, adaptive attention weights can in principle be visualized to highlight atoms or sub—structures considered critical for prediction, providing a way to bridge the “black box” gap. More specifically, the generalization ability of our model has been rigorously validated. The use of scaffold—based splitting ensures that high performance is not the product of evaluations of similar—structure molecules. Moreover, our uncertainty quantification analysis actively identifies predictions with low confidence (high entropy), which usually corresponds to new scaffold or fuzzy situations. This self—awareness enables the model to mark its own limitations. The strong correlation between low prediction entropy and high accuracy (accuracy of 98.5% for H < 0.3, Table 9 in S1 File) validates this intrinsic confidence as a reliable indicator of generalizability of new chemical types.

Finally, this study addresses the gap between computational predictions and wet laboratory validation, laying the foundation for more integrated workflows. MLGT not only provides binary predictions, but also provides calibration probabilities and uncertainty estimates, resulting in a prioritized list of candidates. Highly active, low—uncertainty compounds can be quickly tracked for experimental validation, while high—uncertainty compounds mark areas that require centralized testing or model improvement. This transforms the model from a passive filter to an active component in an iterative design—test—learning cycle, directly contributing to resource optimization in downstream R&D. Although the MLGT framework includes an attention mechanism that can assign important weights to specific atoms, establishing a strict correlation between these weights and chemical intuitions remains a complex challenge that requires specialized case studies.

We recognize that exceptionally high performance in computational screening can sometimes stem from inadvertent data leakage or insufficiently challenging evaluation splits. To preclude this, our study implemented a scaffold—based splitting method, which is widely regarded as a rigorous benchmark in the field of molecular machine learning. Additionally, we employed multiple regularization strategies (e.g., label smoothing, dropout, early stopping) to curb overfitting. The consistent performance gains observed in ablation studies further corroborate that improvements are attributable to model innovations rather than dataset artifacts. Moving forward, we advocate for cross—dataset validation using independent external sources (e.g., BindingDB, PubChem) to further stress—test the model’s generalizability.

In summary, while AI—driven screening holds immense potential for enhancing Uveitis drug discovery, breakthroughs are needed in molecular representations, data quality, model interpretability, and cross—platform validation. Future research should focus on integrating multimodal data (e.g., protein structures, cellular phenotypes), developing highly interpretable architectures, and establishing standardized, high—quality bioactivity databases. These advances will enable truly computationally guided, efficient, and precise drug screening.

### 6.2. Our architecture’s positioning in the field of drug screening

The multimodal MLGT model architecture network based on GATv2 developed in this study is not intended to replace traditional experiments, but rather to serve as an efficient, accurate, and easily interpretable computational filter in the upstream stage of drug discovery. Positioned at the initial stage of the R&D process, the model aims to rapidly screen a vast virtual chemical space and identify a small number of highly promising candidate compounds. This allows subsequent time—consuming and labor—intensive wet experiments to focus on compounds with the highest probability of success, thereby fundamentally optimizing the R&D process.

The MLGT framework is built around GATv2 and multimodal fusion, transcending the traditional role of purely predictive filters in virtual filtering. Its true significance lies in embodying a paradigm shift, from descriptor—dependent black box predictions to learning—representation—driven explainable designs. This section elucidates the fundamental advances that place our work at the forefront of next—generation AI—assisted drug discovery.

First, MLGT redefines molecular representations by unifying topological perception granularity and global chemical intuition. Traditional virtual selection filters oscillate between two extremes: expert—made descriptors (such as ECFP) that capture heuristic knowledge but lack topological fidelity, and pure—map neural networks that learn structures but often ignore overall physicochemical principles. MLGT‘s two—stream fusion is not a simple chain reaction, but a principled reconciliation of these perspectives. The GATv2 trunk acts as a “structural microscope” that dynamically identifies key pharmacological patterns and long—range atomic dependencies, which are often overlooked by fixed fingerprints. At the same time, the descriptor branch acts as a “chemical compass” that directly embeds basic drug similarity rules (such as the Lipinski parameter) into learning goals. This synergy reflects the dual reasoning of drug chemists and establishes a new standard for context—perceived molecular embedding, where local interactions are essentially dependent on global properties.

Second, the framework introduces a path to intrinsically explainable, hypothesis—generated artificial intelligence. While complete explainability remains a challenge, MLGT‘s dynamic attention mechanisms provide a native interface for mechanistic exploration. Unlike opaque deep learning models or static GNNs, adaptive attention weights in GATv2 can be visualized to highlight the contribution of atomic and key levels to prediction. This transforms the model from a black box scorer into a computational test capable of proposing testable structure hypotheses. For example, high—attention graviton structures may be directly related to known binding motifs, or provide new drug efficacy groups for the Uveitis target. Therefore, MLGT shifted the target from passive activity prediction to active SAR (structure—activity relationship) elucidation, bridging artificial intelligence output and drug chemistry insights in a way that could not have been achieved previously using classical QSAR or standard GNN.

Third, our architecture establishes a scalable, end—to—end, accurate treatment screening blueprint. The efficiency of MLGT—screening 300,000 compounds in minutes—demonstrates that complex multimodal learning can be computationally applicable to ultra—large libraries. More importantly, its design is inherently scalable. The fusion module can easily accommodate additional data modalities, such as 3D conformational synthesis, protein targeting maps, or transcriptome characteristics. This makes MLGT not only a fixed tool for Uveitis, but also a core engine for future integrated discovery platforms. By embedding attention—driven interpretability, multimodal flexibility, and high throughput capabilities into a single framework, our work provides the architectural foundation for a closed—loop “design—prediction—analysis” system, ultimately accelerating the transition from heuristic screening to rational, AI—driven treatment design.

Moreover, incorporating dynamic attention mechanisms into GATv2 provides a theoretical advantage in terms of model transparency compared to traditional “black box” deep learning methods. Unlike standard messaging networks that treat all neighbors equally, GATv2 explicitly computes attention coefficients for atomic pairs. Although the detailed visualization and pharmacological validation of these attention weights exceed the scope of this study, this architecture essentially provides the mathematical basis for future interpretability analyses. Theoretically, these attention weights can be used to identify highly important atomic structures, potentially helping pharmacologists isolate the drug clusters that drive predictive biological activity. We position this architecture as the basis on which further granularity analysis can facilitate more explicable “dialogues” between computational predictions and drug chemical intuitions.

In conclusion, MLGT‘s contribution is both methodological and conceptual. It provides the most advanced predictive performance, while driving the field towards a more explainable, integrated, and scalable computational discovery paradigm. MLGT‘s success highlights a key future direction: the most influential AI models in drug discovery will be those that can not only predict, but also explain, generalize, and guide.

### 6.3. Future improvements

Looking ahead, breakthroughs in drug screening will come from transitioning models from passive filters to active design engines. This necessitates going beyond property prediction to deeply integrate with generative AI, thereby establishing a closed—loop “generation–evaluation–optimization” system. For example, one could combine our predictive model with a generative model (such as a GAN or diffusion model) using the predictor as a reward function. The system could proactively explore vast chemical spaces and generate novel molecules that optimize predicted activity and desirable ADMET properties, achieving truly de novo drug design. Furthermore, incorporating uncertainty quantification methods (e.g., Monte Carlo dropout or deep ensembles) will be crucial for providing confidence estimates with each prediction. This would allow prioritizing the most promising candidates for experimental validation, maximizing information gain from limited resources.

At the architectural level, future improvements will focus on rigorously validating the interpretability of the attention weights. We aim to conduct case studies visualizing high—attention atomic regions and correlating them with established structure—activity relationship (SAR) data to transform the model’s predictive power into explanatory insight. Current 2D graph representations are efficient but inherently ignore three—dimensional conformations and flexibility, which are essential for molecule–target interactions. Next—generation models should integrate 3D isometric graph neural networks capable of directly processing atomic coordinates and handling rotations and translations invariantly, thus more realistically simulating binding modes. Similarly, simple feature concatenation is not optimal for multimodal fusion; we envision introducing cross—modal attention mechanisms to enable dynamic, targeted interactions between graph—derived atomic features and global descriptor vectors. This could capture complex nonlinear relationships between topology and macroscopic properties more effectively.

To further enhance the generalizability and physical relevance of MLGT in anti-glycinitis virtual screening, we plan to systematically introduce and compare several cutting-edge map learning paradigms in future work, and propose verifiable experimental routes based on this paper: (1) 3D isometric geometric GNNs (E(n)/SE(3)-equivalent): spatial symmetry can be directly modeled with long-range electrostatic/van der Waar drug interaction by utilizing molecular 3D conformational information (based on RDKit/ETKDG or high-precision energy minimization) and adopting isometric networks [34], thereby enhancing discriminatory ability towards stereochemistry and binding conformational sensitivity; (2) Graph-transformers and global attention mechanisms: combining transformer-type global attention (e.g., Graphormer style) with local GATv2 layers to capture global interaction patterns across distant topological distances in molecules, thereby compensating for limitations of local messaging; (3) subgraph/motif-aware (subgraph/motif-aware) based high-order GNNs and interpretability enhancements: enhancing recognition and interpretability of drug effect groups by constructing drug effect-related chemical substructures (pharmacophores, circumscribed and substitution base patterns) and explicitly modelling them in models; (4) self-supervised/comparative pre-training and cross-task fine-tuning: pre-training map coders (GraphCL/Masked-node, etc.) on a large-scale unlabeled compound repository, before fine-tuning Uveitis tasks to mitigate the generalization bottlenecks caused by small sample labeling scarcity; (5) isometric map and knowledge map fusion: combining molecular maps with isometric entities such as protein/pathway/drug interaction via HIN (heterogeneous information network) or map attention, promising to combine pharmacological a priori with structural information for more accurate candidate screening [35].

This study will conduct step-by-step implementation and systematic comparison of the above methods (same data segmentation, unified evaluation metrics, and statistical tests) in subsequent studies, and will report for each method: 1 performance improvement (AUC, F1, recall, etc.) and significance test (DeLong/ bootstrap), 2 model calculation cost (parameter quantity, training/inference time, and memory), and 3 interpretability analysis (attention/SHAP/subgraph importance). Through this repeatable, quantifiable comparison scheme, it is possible to transform the “potential of the new method” from theoretical inference to falsifiable experimental conclusions.

Ultimately, our vision is to build a universal molecular agent that transcends single—task prediction. This requires pre—training a large molecular graph encoder via self—supervised learning on massive unlabeled chemical databases, to acquire rich chemical knowledge. Then, efficient downstream fine—tuning can be performed for specific tasks like Uveitis screening. This “pre—training–fine—tuning” paradigm would significantly alleviate the bottleneck of scarce bioactivity data. In summary, the future evolution of this architecture represents a comprehensive upgrade from algorithmic core to system design, elevating AI from an auxiliary tool to a core engine driving drug discovery. Through deeper data mining, more physically realistic models, and closed—loop system design, it ultimately aims to achieve a step—change in R&D efficiency.

## Conclusion

This study develops a novel graph neural network framework, MLGT, which combines GATv2 with multimodal molecular representation to address the key challenge of efficiently and accurately screening candidate drugs for Uveitis. By synergistically combining molecular graph topology, bond features, and physicochemical descriptors within a unified deep learning architecture, our model achieves state—of—the—art prediction performance, significantly outperforming existing methods in accuracy, recall, and F1 score on rigorously selected datasets. The proposed framework not only effectively captures complex structure—activity relationships but also alleviates the data imbalance problem and enhances the model’s generalization ability through advanced training strategies such as label smoothing, class—balanced sampling, and dynamic attention mechanisms.

This work provides a robust computational foundation for accelerating drug discovery in ophthalmology and other complex immune—mediated diseases. By offering a highly accurate AI—driven screening tool embedded with attention mechanisms, our approach reduces reliance on costly and time—consuming wet—lab experiments and enables rapid identification of promising therapeutic candidates from large chemical libraries. The integration of multimodal features and the potential for attention—based analysis offers a pathway to bridge the gap between computational prediction and medicinal chemistry intuition between computational prediction and medicinal chemistry intuition, facilitating more informed decision—making in early—stage drug development [36].

While the integration of graph features and molecular descriptors is not unprecedented, MLGT advances the field through:Dynamic attention—based fusion rather than static concatenation;Descriptor—informed graph pooling that conditions graph readout on global molecular properties;Task—specific regularization tailored to imbalanced, small—scale bioactivity data;Explicit modeling of bond attributes alongside atom features within the GATv2 framework.These design choices enable MLGT to capture complex structure–activity relationships specific to Uveitis, a disease with multifactorial immune pathology that demands more nuanced molecular representations.

Looking forward, the proposed framework has substantial potential for broader applications in targeted therapy development for other diseases. Future work will focus on incorporating three—dimensional molecular conformations, integrating protein–target interaction data, and extending the model to generative design tasks for de novo molecule generation. Scaling the system to handle ultra—large virtual libraries and validating predictions through experimental assays will be critical for clinical translation. This study underscores the transformative role of artificial intelligence in advancing precision medicine and sets a new benchmark for computational drug screening methodologies.

## Supporting information

S1 File Raw image. (PDF)
