# Supplementary material for: MLGT: A multimodal graph attention network for virtual screening of anti—Uveitis drugs
Source: PLoS One. 2026 Mar 5;21(3):e0343159. doi: 10.1371/journal.pone.0343159 (PMC12962487; doi:10.1371/journal.pone.0343159)
Supplement: S1 File — (PDF) [file pone.0343159.s001.pdf]

Table 1. Research Differences

| Category | Limitations | Research Gap | Strengths of This Study |
|---|---|---|---|
| Model Architecture | Static Attention Mechanism | Fixed attention scoring function fails to adapt to complex atomic interactions. | Dynamic attention (GATv2) ena bles adaptive weighting of ato mic interactions, enhancing sen sitivity to key functional group s and non—local effects. |
| Feature Representation | Single—source origin | Underutilization of multimodal molecular information. | Multimodal fusion integrates gr aph topology with physicoche mical descriptors, providing a holistic representation that imp roves predictive accuracy and model interpretability. |
| Training Strategy | The training process is straightforward. | Poor generalization on minority classes in bioactivity data. | Advanced regularization (label smoothing, class—balanced sa mpling) mitigates overfitting a nd improves calibration, especi ally for rare active compounds. |
| Task—specific | Direct application of the general model | Models not tailored to multi —target immune mechanisms of Uveitis. | Disease—aware customization f rom data curation to feature se lection enhances relevance to Uveitis drug screening and im proves real—world applicabilit y. |
| Data Utilization | Sensitive to sparse data | Limited labeled data hinders model performance. | Data augmentation via SMILES randomization expands trainin g diversity without additional experimental cost, improving r obustness on low—resource da tasets. |

Table 2. Representative advanced AI paradigms in recent drug discovery studies and their relationship to MLGT.

| Paradigm | Core idea | Representative studies | Relation to MLGT |
|---|---|---|---|
| LLM + Knowledge Graph | Encode biomedical text and structured knowledge via LLMs and KG reasoning | LLM-DDI, DDI-GPT–style frameworks | Provides external domain knowledge; complementary to MLGT's chemistry-centric modeling |
| Multi-view contrastive learning | Align molecular graphs, fingerprints, and networks via contrastive objectives | Multi-view contrastive DDI models | Motivates robust multimodal representation learning under data scarcity |
| Graph self-supervised pretraining | Pretrain GNNs using contrastive or masked objectives | GraphCL, DeepGCL variants | Potential pretraining strategy for MLGT graph encoders |
| Multimodal fusion models | Combine structure and global descriptors | Recent multimodal drug screening models | Core design principle of MLGT |

Table 3. Components Related to the Dataset

| Dataset | Total number of compounds | Number of positive compounds | Number of negative compounds |
|---|---|---|---|
| Train | 250000 | 75000 | 175000 |
| Dev | 40000 | 12000 | 28000 |
| Test | 10000 | 3000 | 7000 |

Table 4. Experimental Environment

| Experimental Environment | Configuration |
|---|---|
| Operating System | Windows 11 |
| CPU | Intel(R) Core(TM) i7–10400 CPU @ 2.90 GHz 2.90 GHz |
| GPU | NVIDIA GeForce RTX 5060x2 |
| Memory | 256 GB |
| Python | 3.8.0 |

Table 5. Parameters

| Model Parameter | Value |
|---|---|
| **gat_hidden_channels** | 256 |
| **gat_num_layers** | 3 |
| **gat_num_heads** | 8 |
| **gat_concatenate** | True |
| **atom_feature_dim** | 78 |
| **bond_feature_dim** | 12 |
| **output_size** | 2 |
| **ffn_hidden_size** | 512 |
| **ffn_num_layers** | 2 |
| **ffn_dropout** | 0.2 |
| **batch_size** | 128 |
| **epochs** | 200 |
| **learning_rate** | 2e−4 |
| **weight_decay** | 1e−6 |
| **early_stopping_patience** | 20 |
| **gradient_clip** | 3.0 |
| **accumulation_steps** | 2 |
| **label_smoothing** | 0.1 |
| **t_mult** | 2 |
| **Random seed** | 42 |

Table 6. Comparative Experimental Analysis

| Model | Accuracy(%) | Recall(%) | F1(%) |
|---|---|---|---|
| **GCN** | 93.1 | 91.5 | 91.1 |
| **GAT (standard)** | 94.8 | 92.7 | 93.1 |
| **Attentive FP** | 91.6 | 89.2 | 89.9 |
| **Chemprop** | 89.7 | 88.3 | 89.2 |
| **RF—SMILES** | 94.2 | 92.4 | 93.4 |
| **MLGT (Ours)** | 97.7 | 96.1 | 97.2 |

Table 7. Comparative Experimental Analysis

| Model | Accuracy(%) | Recall (%) | F1(%) |
| --- | --- | --- | --- |
| Ablation of Attention Pooling Module | 92.8 | 90.2 | 90.4 |
| Ablation of Molecular Descriptors | 92.1 | 88.9 | 89.7 |
| Ablation of GATv2 (use GAT) | 94.8 | 92.7 | 93.1 |
| Ablation of Class—Balancing Sampling | 89.6 | 88.9 | 90.1 |
| Ablation of Label Smoothing | 93.3 | 93.2 | 92.6 |
| MLGT (Ours) | 97.7 | 96.1 | 97.2 |

Table 8. MLGT Model Complexity Analysis

| Components | Number of Parameters （M） | Computation Complexity | Memory Occupation (Training)(MB) | Memory Occupation (Reasoning)(MB) |
|---|---|---|---|---|
| GATv2 Backbone Network | 2.8 | O(L × H × (N × d² + E × d)) | 420 | 85 |
| — Level 1 (input: 78→256) | 1.2 | O(8 × (N×78× 256 + E×256)) | 160 | 32 |
| — Level 2—3 (256→256) | 1.6 | O(16 × (N×256² + E×256)) | 260 | 53 |
| Descriptor Processing Networks | 0.2 | O(50×64 + 64×32) | 40 | 8 |
| Classifier (FFN) | 0.5 | O(544×512 + 512× 2) | 100 | 20 |
| Total Model | 2.8 | O(3×8×(N×256² + E×256)) | 560 | 113 |
| Average per molecule | — | O(N≈50, E≈60) → ~0.2M FLOPs | — | ~0.4KB |

Table 9. Model performance stratified by prediction uncertainty

| Entropy Range | Accuracy(%) | Recall(%) | F1(%) |
|---|---|---|---|
| H<0.3 | 98.5 | 97.8 | 98.1 |
| 0.3≤H<0.6 | 95.2 | 94.6 | 94.9 |
| H>0.6 | 76.2 | 75.4 | 74.8 |

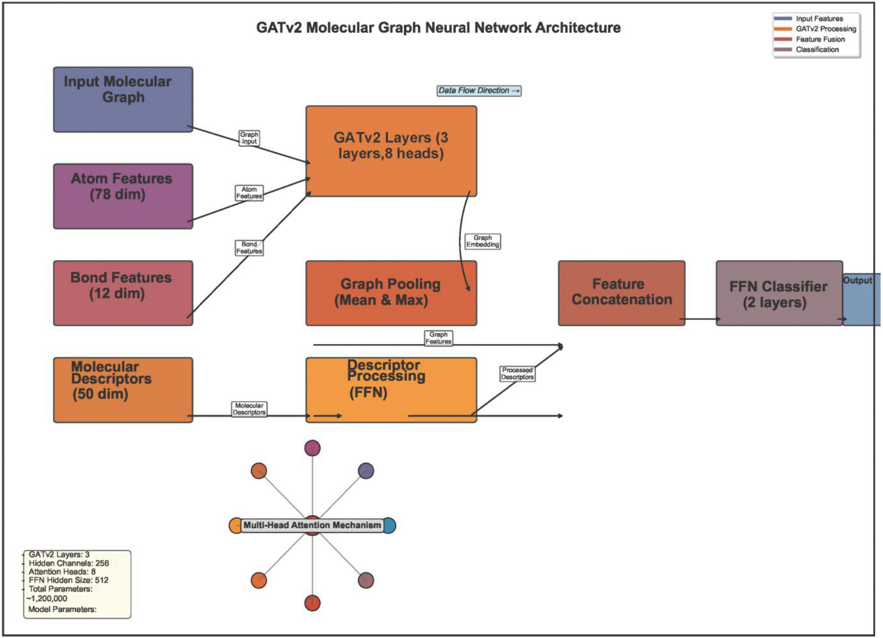


Figure 1. Model Architecture Diagram

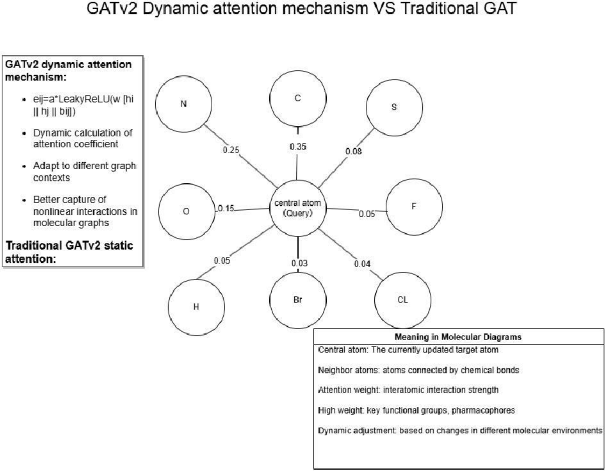


Figure 2. GATv2 Dynamic attention mechanism VS Traditional GAT

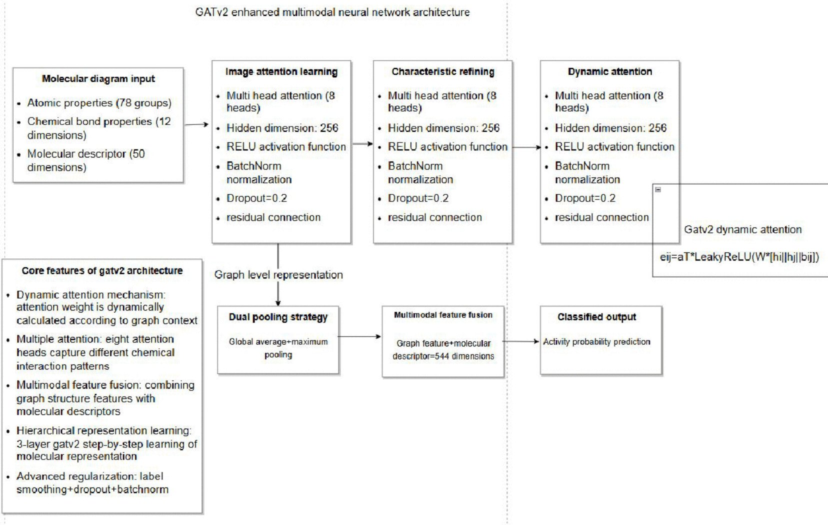


Figure 3. Gatv2 enhanced multimodal neural network architecture

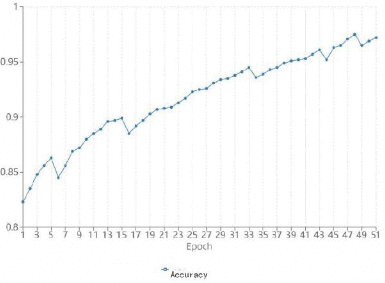


Figure 4. Accuracy

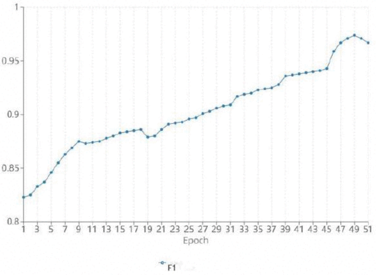

1
0.95
0.9
0.85
0.8
1 3 5 7 9 11 13 15 17 19 21 23 25 27 29 31 33 35 37 39 41 43 45 47 49 51
Epoch
F1


Figure 5. F1

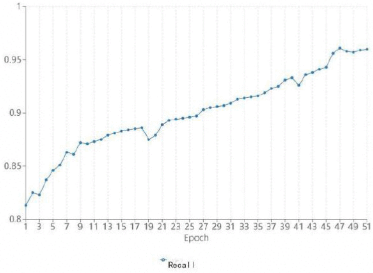


Figure 6. Recall

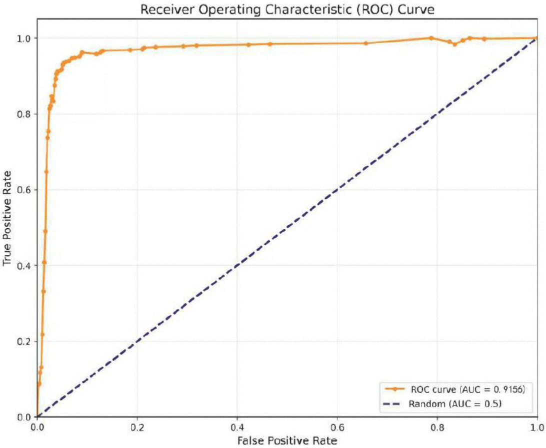


Figure 7. AUC−ROC curve

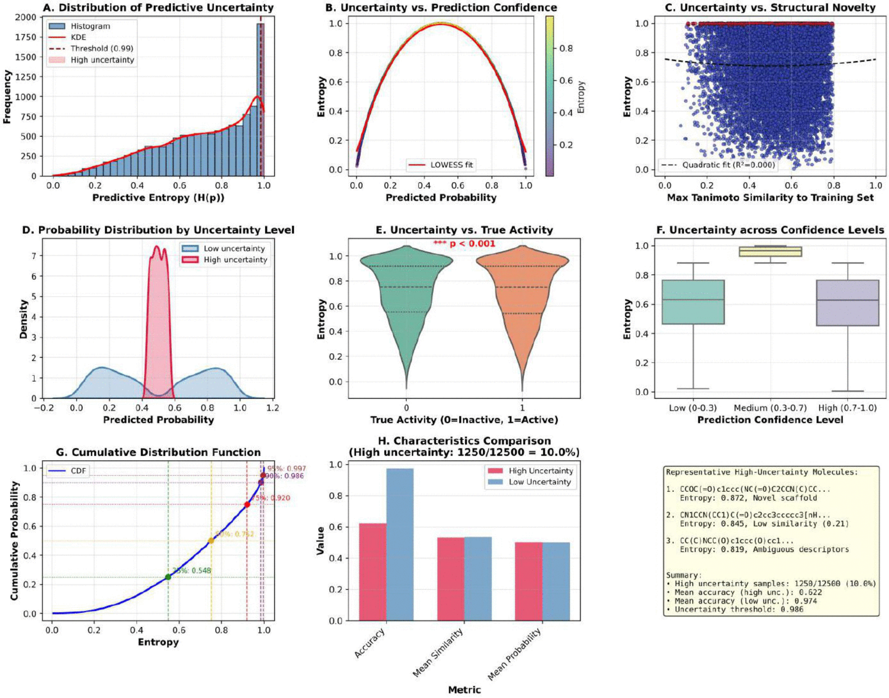


Figure 8. Uncertainty Analysis
